# Supplementary material for: Content-rich biological network constructed by mining PubMed abstracts
Source: BMC Bioinformatics. 2004 Oct 8;5:147. doi: 10.1186/1471-2105-5-147 (PMC528731; doi:10.1186/1471-2105-5-147)
Supplement: Additional File 2 — The original results of the above study (non-essential files are deleted to keep the file size under the limit set by BMC bioinformatics). [file 1471-2105-5-147-S2.bz2 › chilibotAdditionalFile2/dip10/72ID2005966E280/html/RBBP7_RB1.html]

 


 **RBBP7** and **RB1** 
  
Found 11 abstracts in PubMed, retrieved 10.  
 

 What does Google say? 
 PDF only 
| .edu only 

---

**Interactive relationship** (e.g. stimulation, inhibition, etc)

**Non-interactive relationship** (e.g. studied together, co-existance, homology, etc.)

- The levels of expression of activating transcription factor 3, prefoldin 4, and retinoblastoma  [ **RB1** ]  binding protein 7  [ **RBBP7** ]  significantly increased in accordance with that of BCL6, suggesting that Bcl 6 may act as a transcriptional activator.  Ref: 12414651 Cancer Res, 2002
- The gene was identified as retinoblastoma  [ **RB1** ]  suppressor Rb associated protein 46 RbAp46  [ **RBBP7** ] , a nuclear protein that interacts physically with Rb and is a component of the human mSin3 co repressor complex.  Ref: 9765217 J Biol Chem, 1998
- Unlike the other maize deacetylases, HD1A and nucleolar HD2, HD1B copurified as a complex with a protein related to the retinoblastoma associated protein  [ **RB1** ] , Rbap46  [ **RBBP7** ] .  Ref: 10677216 Biochemistry, 2000
- The retinoblastoma  [ **RB1** ]  suppressor Rb associated protein 46 RbAp46  [ **RBBP7** ]  is a nuclear protein of the WD repeat protein family and a component of the histone deacetylase complex that physically interacts with Rb.  Ref: 11433396 Int J Cancer, 2001
- We have found previously that the retinoblastoma  [ **RB1** ]  Rb suppressor associated protein 46 RbAp46  [ **RBBP7** ]  is a gene upregulated by the Wilms tumor suppressor, WT1, and functions as a potent growth inhibitor.  Ref: 12767060 Int J Cancer, 2003
- Characterization of the gene encoding mouse retinoblastoma  [ **RB1** ]  binding protein 7  [ **RBBP7** ] , a component of chromatin remodeling complexes.  Ref: 12376095 Genomics, 2002
- The retinoblastoma  [ **RB1** ]  suppressor Rb associated protein 46 RbAp46  [ **RBBP7** ]  is a member of the WD repeat protein family and a component of the histone modifying and remodeling complexes.  Ref: 11394910 Biochem Biophys Res Commun, 2001
